# Systematic volumetric analysis predicts response to CSF drainage and outcome to shunt surgery in idiopathic normal pressure hydrocephalus

**DOI:** 10.1007/s00330-020-07531-z

**Published:** 2021-01-03

**Authors:** Dan Wu, Abhay Moghekar, Wen Shi, Ari M. Blitz, Susumu Mori

**Affiliations:** 1 Key Laboratory for Biomedical Engineering of Ministry of Education, Department of Biomedical Engineering, College of Biomedical Engineering & Instrument Science, Zhejiang University, Hangzhou, 310027 Zhejiang China; 2 Department of Neurology, Johns Hopkins University School of Medicine, Baltimore, MD 21205 USA; 3 Department of Radiology, University Hospitals, Case Western Reserve University, Cleveland, OH 44106 USA; 4 F.M. Kirby Research Center for Functional Brain Imaging, Kennedy Krieger Institute, Baltimore, MD 21205 USA

**Keywords:** Hydrocephalus, Normal pressure, Segmentation, Volume, Algorithm

## Abstract

**Objectives:**

Idiopathic normal pressure hydrocephalus (INPH) is a neurodegenerative disorder characterized by excess cerebrospinal fluid (CSF) in the ventricles, which can be diagnosed by invasive CSF drainage test and treated by shunt placement. Here, we aim to investigate the diagnostic and prognostic power of systematic volumetric analysis based on brain structural MRI for INPH.

**Methods:**

We performed a retrospective study with a cohort of 104 probable INPH patients who underwent CSF drainage tests and another cohort of 41 INPH patients who had shunt placement. High-resolution T1-weighted images of the patients were segmented using an automated pipeline into 283 structures that are grouped into different granularity levels for volumetric analysis. Volumes at multi-granularity levels were used in a recursive feature elimination model to classify CSF drainage responders and non-responders. We then used pre-surgical brain volumes to predict Tinetti and MMSE scores after shunting, based on the least absolute shrinkage and selection operator.

**Results:**

The classification accuracy of differentiating the CSF drainage responders and non-responders increased as the granularity increased. The highest diagnostic accuracy was achieved at the finest segmentation with a sensitivity/specificity/precision/accuracy of 0.89/0.91/0.84/0.90 and an area under the curve of 0.94. The predicted post-surgical neurological scores showed high correlations with the ground truth, with *r* = 0.80 for Tinetti and *r* = 0.88 for MMSE. The anatomical features that played important roles in the diagnostic and prognostic tasks were also illustrated.

**Conclusions:**

We demonstrated that volumetric analysis with fine segmentation could reliably differentiate CSF drainage responders from other INPH-like patients, and it could accurately predict the neurological outcomes after shunting.

**Key Points:**

*• We performed a fully automated segmentation of brain MRI at multiple granularity levels for systematic volumetric analysis of idiopathic normal pressure hydrocephalus (INPH) patients.*

*• We were able to differentiate patients that responded to CSF drainage test with an accuracy of 0.90 and area under the curve of 0.94 in a cohort of 104 probable INPH patients, as well as to predict the post-shunt gait and cognitive scores with a coefficient of 0.80 for Tinetti and 0.88 for MMSE.*

*• Feature analysis showed the inferior lateral ventricle, bilateral hippocampus, and orbital cortex are positive indicators of CSF drainage responders, whereas the posterior deep white matter and parietal subcortical white matter were negative predictors.*

**Supplementary Information:**

The online version contains supplementary material available at 10.1007/s00330-020-07531-z.

## Introduction

Idiopathic normal pressure hydrocephalus (INPH) is characterized by the clinical triad of dementia, gait dysfunction, and urinary incontinence due to excess cerebrospinal fluid (CSF) buildup in the brain. In the absence of any proven medical therapy, surgical placement of a shunt to drain excess CSF is shown to be an effective treatment [1, 2], although controversy remains [3]. Diagnosis of INPH, however, is challenging because its clinical and radiological presentations also occur in aging, cerebrovascular disorders, neurodegenerative diseases, and other forms of hydrocephalus [4]. Currently, one of the most effective tests is CSF removal by a large volume lumbar puncture (LVLP) or extended CSF drainage by lumbar drainage (ELD) [4]. Temporal improvement after CSF drainage is known to predict shunt response, but these invasive tests could cause variable complications [5]. The feasibility of using non-invasive neuroimaging tools, i.e., MRI, to replace or complement the invasive test has been recently investigated [6–9].

MRI features of INPH involve ventricular enlargement, steep callosal angle, Sylvian fissure expansion, disproportionately enlarged subarachnoid space hydrocephalus (DESH), and cerebral atrophy [10]. These features are often assessed by visual inspection or semi-quantitative measures such as Evan’s index [11] or DESH scale [10], which, however, are not sufficient in themselves to establish a diagnosis [12]. Quantitative analysis was also attempted. Volumetric analysis based on segmentation of gray matter (GM), white matter (WM), and ventricles provided reasonable diagnostic accuracy in separating INPH from Alzheimer’s disease (AD), Parkinson’s disease (PD), or healthy controls [13–15]. CSF distribution was also proposed as a useful marker of INPH [16, 17]. Yet, the comparison between INPH and normal elderly, AD, or PD may not address the key clinical challenge, as these diseases are known to have distinctive radiological and clinical signs. Moreover, the neurological outcomes after shunting are variable [18], but the predictive value of pre-operative MRI remains moderate [19–22]. For instance, a recent large cohort study showed that the accuracy in predicting favorable shunt outcomes was only 0.58, even with comprehensive multi-modal markers [8].

The previous volumetric studies of INPH used coarse tissue segmentation of the GM, WM, and CSF, or examined single brain region based on certain hypotheses, but did not systematically evaluate the whole-brain structural volumes and their distribution. In this study, we aim to evaluate the full potential of volumetric analysis in the diagnosis and prognosis of INPH, using an automatic whole-brain segmentation pipeline [23] that parcellates the brain into 283 structures and allows volumetric analysis at multiple levels of granularity [24]. We hypothesize that the volumetric markers could accurately predict the response to CSF drainage in NPH-like patients and predict the neurological outcome after shunt surgery in confirmed NPH patients.

## Materials and methods

### Patients

Patient data were retrospectively collected between the years of 2009 and 2016. All research protocols were approved by the local Institutional Review Board. Written informed consent was obtained from all patients in this study. Two patient cohorts were included in this study:

1. Probable INPH were included to test the diagnostic accuracy of separating responders to CSF drainage from non-responders. Inclusion criteria were as follows: (*i*) a diagnosis of probable INPH based on routine radiological reports, characteristic gait, and cognitive symptoms; (*ii*) completion of gait assessments before and within 2 h after the CSF drainage test via LVLP; and (*iii*) completion of MRI scans between 1 and 3 months prior to the test. Patients with obstructive hydrocephalus, congenital hydrocephalus, or secondary hydrocephalus were excluded. One hundred four patients were selected according to these criteria, with their demographic and clinical information summarized in Table 1. Thirty-five among them were identified as responders who presented an improvement on the timed up and go (TUG) test by 30% or Tinetti gait assessment by 5 or more points within 2 h after drainage compared to pre-drainage assessments.
2. A separate cohort of INPH patients was used to test the prognostic accuracy of post-surgical outcomes because we did not have follow-up neurological tests of the responders from the previous cohort. We selected patients who completed Tinetti and/or Mini-Mental State Examination (MMSE) tests before shunting and within 1 year after shunting and had MRI scans between 0 and 10 months prior to shunting. The exact interval between the tests and shunt surgery and between surgery and MRI scans are provided in Table 2. Forty-seven patients who had complete Tinetti records and 37 patients who had complete MMSE records were included (Table 2).

**Table 1:** Basic demographic and clinical information of the patients used in the diagnostic test (cohort 1) and their gait and cognitive assessments before and after the CSF drainage test. Unless specified, results are reported as mean (standard deviations)

	Responders (*n* = 35)	Non-responders (*n* = 69)	*p* (responder vs non-responders)	*p* (pre- vs post-CSF drainage)
Age	74.0 (8.4)	73.5 (10.5)	0.8144 ^a^	
Male, *n* (%)	20 (57.1%)	39 (56.5%)	0.8815 ^b^	
Evan’s index	0.365 (0.035)	0.372 (0.052)	0.0133 ^c^	
Pre-test Tinetti	13.43 (5.94)	15.23 (5.40)	0.0947 ^a^	< 0.0001 ^d^ (responder) /0.0056 ^d^ (non)
Post-test Tinetti	18.14 (6.08)	17.97 (5.23)	0.7578 ^a^	
Pre-test TUG	25.68 (19.71)	16.97 (13.10)	0.0988 ^a^	< 0.0001 ^d^ (responder) /< 0.0001 ^d^ (non)
Post-test TUG	20.80 (20.18)	17.09 (12.24)	0.9924 ^a^	
Pre-test MOCA	20.28 (4.78)	20.49 (4.96)	0.6963 ^c^	< 0.0001 ^d^ (responder) /< 0.1481 ^d^ (non)
Post-test MOCA	22.31 (4.59)	21.04 (4.10)	0.2135 ^c^	

^a^Wilcoxon rank-sum test; ^b^Chi-square test; ^c^Unpaired *t* test with unequal variance; ^d^Wilcoxon matched-pairs signed-rank test

**Table 2:** Basic demographic and clinical information of the patients used in the prognostic test (cohort 2), their MMSE and Tinetti assessments before and after shunting, and the time (in days) between shunt surgery and post-surgical test. Unless specified, results are reported as mean (standard deviations)

Type of test	Male, *n* (%)	Age	Score before shunting	Score after shunting	Days after shunting
Tinetti (*n* = 47)	23 (57.5%)	72.6 (7.0)	18.9 (5.2)	22.7 (5.5)*	83.2 (54.3)
MMSE (*n* = 37)	18 (58.1%)	74.6 (5.7)	25.3 (4.4)	25.2 (4.7)	142.4 (85.9)

*p < 0.0001 by Wilcoxon matched-pairs signed-rank test to compare the scores before and after shunt surgery

### Neurological examinations

A physical therapist (A.M.) administered the Tinetti Performance Oriented Mobility Assessment [25] and TUG test [26], while a physician (A.D.) or research assistant administered the (MMSE) [27] and the Montreal Cognitive Assessment (MOCA) [28]. The Tinetti is a structured semi-quantitative scale that examines different aspects of gait and balance (range: 0–28). The MMSE is a well-known screening test for cognitive function (range: 0–30). Higher scores on both tests represent better performance.

### MRI acquisition

All patients had 3D high-resolution T1-weighted images scanned on a 3-T Trio or Verio scanner (Siemens). Images were acquired using an MPRAGE sequence with the following imaging parameters: field of view of 240 × 240 mm, in-plane resolution of 0.76–0.94 mm, slice thickness of 0.9–1.2 mm, sagittal orientation, echo time of 2.1–3.25 ms, repetition time of 1800–2110 ms, and flip angle of 8–9°, parallel acceleration factor of 2, and a scan time of approximately 5 min.

### Brain segmentation

**Fig. 1:**
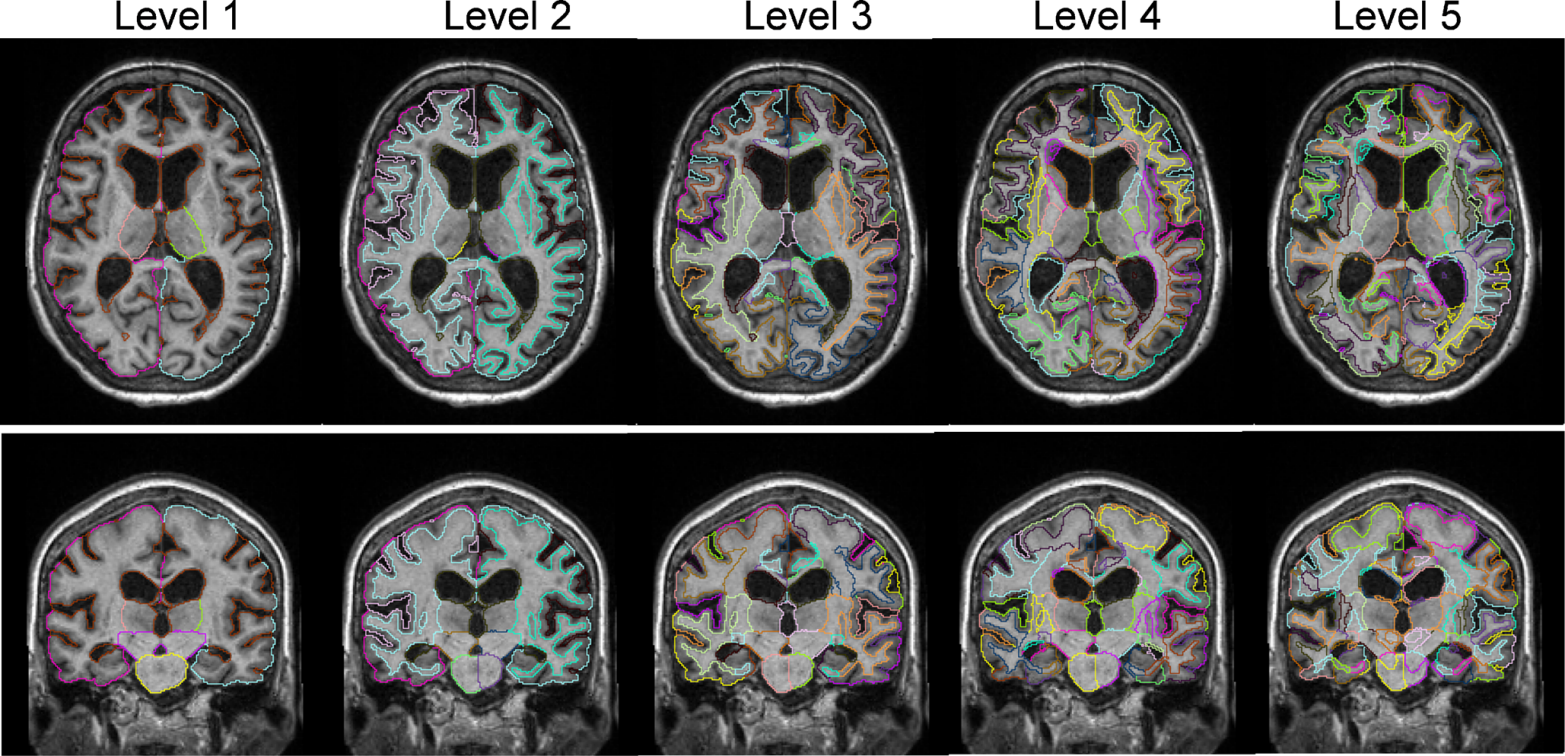
Multi-atlas-based segmentation of the brain of an INPH patient. Structural labels are shown at different granularity levels from level 1 (7 labels) to level 5 (283 labels), in transverse and coronal views

The high-resolution T1-weighted images were segmented using a multi-atlas algorithm [29] via a cloud platform—the MRICloud (www.mricloud.org). Details of the segmentation algorithm were provided in [23]. We used an elderly brain atlas set [30] that was segmented into 283 regions of interest (ROIs), including 96 GM ROIs, 114 WM ROIs, 12 ventricular ROIs, 18 sulci ROIs, 28 brainstem and cerebellar ROIs, and 15 junk labels as place holders (Supplementary Table S1). The 283 ROIs at the finest granularity level were grouped into higher hierarchical levels, according to their ontological relationship [31]. Five granularity levels were defined in the current atlas (Fig. 1), which consisted of 7, 19, 54, 137, and 283 ROIs for levels 1, 2, 3, 4, and 5 [24].

### Feature selection and classification analysis for cohort 1

The discriminative features (ROI volumes) for differentiating responders to CSF drainage were selected using a recursive feature elimination (RFE) method [32] using the scikit-learn toolbox in Python (scikit-learn.org). Age and gender were also included in the model as additional features. A linear support vector machine (SVM) was incorporated into the RFE as the classifier. Class-weighting [33] was used in the SVMs to address the imbalanced sample size between responders and non-responders. We determined the optimal features by performing RFE from one to all possible features at each granularity level and selected the combination that provided the highest classification accuracy at each level. The classification performance was evaluated using a leave-one-out cross-validation scheme and was assessed by sensitivity, specificity, precision, accuracy, and area under the curve (AUC) of the receiver operating curve (ROC).

### Prediction analysis for cohort 2

We used the least absolute shrinkage and selection operator (LASSO) [34] method to select the optimal features and determine the regression model for predicting post-surgical Tinetti and MMSE, in R (www.r-project.org). The ROI volumes at levels 1–4, along with the age, gender, test score before shunting, the time between shunt surgery and post-surgical test, and time between the pre-surgical MRI and shunt surgery, were used as predictors. The regularization factor in LASSO was kept at 0.5 for training. ROI volumes at level 5 were not used because the number of features (*n* = 283) was too large for this cohort (*n* = 37for MMSE and 47 for Tinetti). We used leave-one-out cross-validation to predict the outcomes of individual patients, and correlated the predicted scores with the ground truth.

### Statistical analysis

Differences of the clinical characteristics between the responders and non-responders in cohort 1 were tested using the Wilcoxon rank-sum test for data not satisfying normal distribution (age, Tinetti, and TUG before and after CSF drainage), unpaired *t* test with unequal variance for Evan’s index and MMSE before and after CSF drainage, and chi-square test for gender. The Wilcoxon matched-pairs signed-rank test was used to compare the test scores before and after CSF drainage or shunt surgery in cohort 2. The statistical tests were performed in R.

## Results

### Diagnosis of CSF drainage responders in cohort 1

The demographic and clinical characteristics were equivalent between the responder and non-responder groups (Table 1), except for Evan’s index (*p* < 0.013). Ninety-four percent of the responders and 91.3% of the non-responders had Evan’s index above the threshold of 0.3, which is one of the diagnostic criteria for INPH [35]. Using Evan’s index, age, and gender as features, the diagnostic accuracy was only 0.42 at an optimal threshold of 0.35 with an AUC of 0.40 (Table 3), indicating the challenges of separating responders from the probable INPH patients with traditional markers.

**Table 3:** Classification accuracy of separating responders and non-responders to CSF drainage, using the optimal volumetric features at the different granularity levels of segmentation, as evaluated by the sensitivity, specificity, precision, accuracy, and AUC. The classification performance of Evan’s index was also provided for comparison

Level	#ROI	Optimal #feature	Sensitivity	Specificity	Precision	Accuracy	AUC
Evan’s index	-	-	0.80	0.22	0.35	0.42	0.40
1	7	4	0.66	0.58	0.43	0.60	0.32
2	19	7	0.63	0.67	0.49	0.65	0.41
3	54	5	0.54	0.75	0.53	0.68	0.56
4	137	16	0.71	0.86	0.69	0.80	0.82
5	283	79	0.84	0.91	0.89	0.90	0.94

**Fig. 2:**
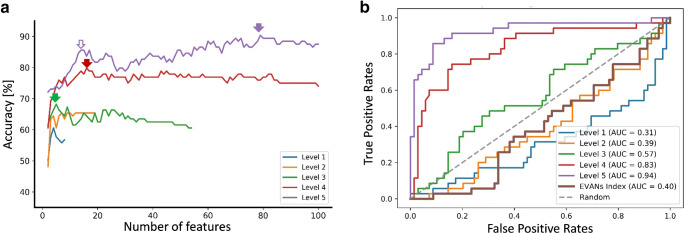
Classification of the CSF drainage responders and non-responders. **a** Classification accuracy at different levels of segmentation with varying number of features. The solid arrows point to the optimal number of features on each curve, and the hollow purple arrow points to a local peak at level 5. **b** Receiver operation characteristic (ROC) of the classifiers with the optimal sets of features at granularity levels 1–5. ROC of Evan’s index is also compared. The area under the curve (AUC) values are denoted correspondingly

Figure 1 shows the five levels of brain segmentation of an INPH patient. ROI volumes were obtained at the different levels separately for the analysis, while the junk labels were discarded. Figure 2a shows the classification accuracy of separating responders and non-responders to CSF drainage, using structural volumes at levels 1–5 at varying numbers of features. The accuracy increased as the granularity level increased as expected. At level 4, the accuracy fluctuated around 0.8 with a peak at a feature number of 16 (red arrow). Accuracy at level 5 further increased to 0.9 with an optimal feature number of 79 (purple arrow). In addition, at level 5, the accuracy curve showed a local peak (hollow purple arrow) with an accuracy of 0.86 and AUC of 0.89 at a feature number of 19 (7% of the total number of ROIs), indicating that majority of the discriminative information was captured by a few selective anatomical features. The ROC curves in Fig. 2b demonstrated the highest AUC of 0.94 at level 5, followed by an AUC of 0.82 at level 4. The sensitivity, specificity, and precision with the optimal feature numbers at each level are listed in Table 3.

**Fig. 3:**
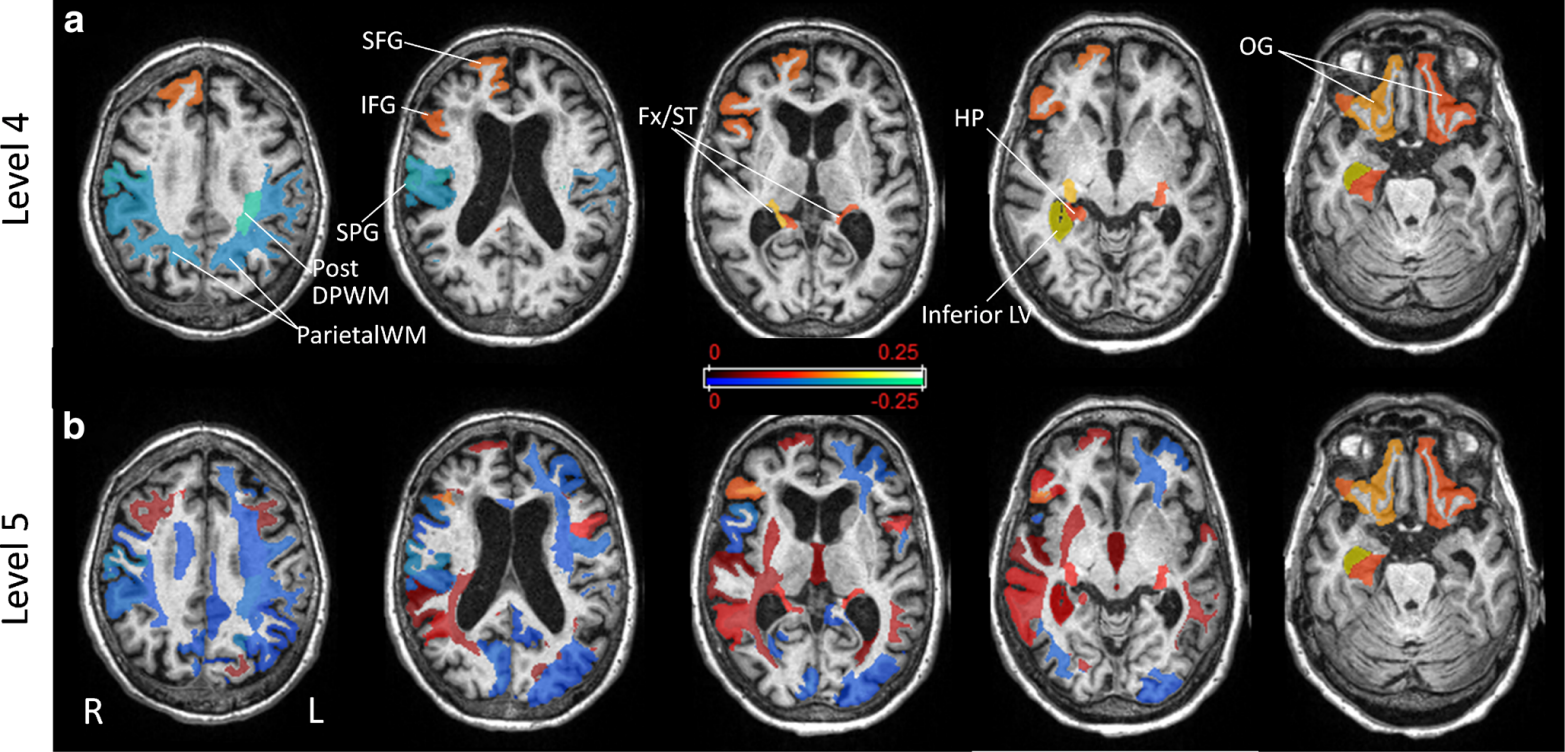
Weight maps of the brain regions that contributed to the classification of the responders and non-responders to CSF drainage at level 4 (**a**) and level 5 (**b**). The color map indicates the weights of selected regions in the RFE model. Abbreviations: post-DPWM, posterior deep white matter; parietal WM, parietal white matter; SFG, superior frontal gyrus; IFG, inferior frontal gyrus; SPG, superior parietal gyrus; Fx/ST, fornix/stria terminalis; HP, hippocampus; inferior LV, inferior lateral ventricle; OG, orbital gyrus

The ROIs selected by RFE and their weights were extracted to characterize the discriminative features in classification. The weight maps in Fig. 3a showed that at level 4, the right inferior lateral ventricle (LV), bilateral fornix/stria terminalis, bilateral orbital gyrus, right superior and inferior frontal gyri, right hippocampus, and cingulum were positive indicators of CSF drainage response, whereas the left posterior deep WM, bilateral parietal subcortical WM, right post-central gyrus, and superior parietal gyral regions were negative indicators. The discriminative features at level 5 shared similarities with those at level 4 (Fig. 3b). The optimal set of ROIs at levels 3, 4, and 5 and their weights were listed in Supplementary Table S2.

### Prognosis of shunt outcome in cohort 2

**Fig. 4:**
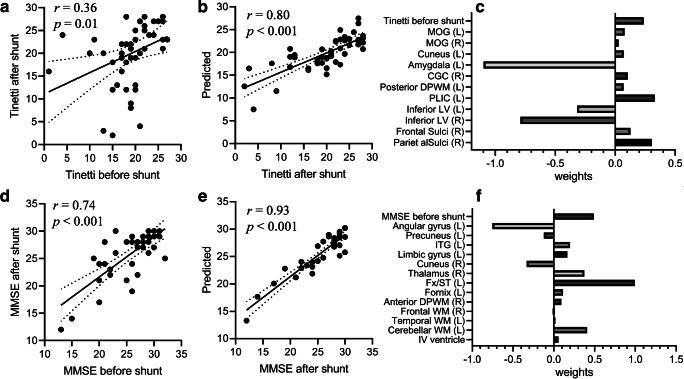
Prediction of Tinetti and MMSE scores after shunting in INPH patients. **a** Correlation between the pre-shunt and post-shunt Tinetti scores in 47 INPH patients. **b** Correlation between the model predicted and clinically measured post-shunt Tinetti scores. **c** Weights of ROIs selected by the LASSO model to predict the post-surgical Tinetti scores. **d** Correlation between the pre-shunt and post-shunt MMSE scores in 47 INPH patients. **e** Correlation between the predicted and measured post-shunt MMSE scores. **f** Weights of ROIs selected by LASSO to predict the post-surgical MMSE scores. Abbreviations: MOG, middle occipital gyrus; CGC, Cingulum (cingulate gyrus part); DPWM, deep WM; PLIC, posterior limb of internal capsule; LV, lateral ventricle; ITG, inferior temporal gyrus; Fx/ST, fornix/stria terminalis

The pre-shunt Tinetti showed a moderate correlation with the post-shunt Tinetti with an *r* of 0.36 (*p* = 0.01, Fig. 4a), and the pre-shunt MMSE showed a relatively high correlation with the post-shunt MMSE (*r* = 0.74 and *p* < 0.001, Fig. 4d), indicating that the baseline neurological performance played an important role in the post-shunt outcome. We then tested the predictive power of volumetric markers at different granularity levels in estimating the gait and cognitive outcomes after shunting, along with the pre-shunt test scores as one of the covariates. The correlations (*r*) between the LASSO predicted Tinetti scores and the clinically measured ground truth were 0.55, 0.56, 0.76, and 0.80 at levels 1, 2, 3, and 4, respectively (*p* < 0.01 for all correlations). The predicted MMSE after shunting was strongly correlated with the ground truth with *r*’s of 0.85, 0.86, 0.87, and 0.88 at levels 1, 2, 3, and 4, respectively (*p* < 0.001 for all correlations). The predicted scores at level 4 were shown in Fig. 4 b and e with respect to the corresponding ground truth. The prediction errors were larger towards lower Tinetti scores because fewer samples were available at low scores. In contrast, the predictions based on Evan’s index and the same covariates showed much lower correlations with the ground truth (*r* = 0.48 for the Tinetti and *r* = 0.80 for MMSE).

We also tested the predictive performance of the LASSO models without the pre-shunt scores. The *r* values at levels 1–4 were 0.53, 0.53, 0.77, and 0.75 for Tinetti and 0.61, 0.75, 0.69, and 0.83 for MMSE, suggesting that the structural volumes alone were also predictive of the post-shunt outcome but combining pre-shunt information could further enhance the performance. Evan’s index–based prediction accuracy was only 0.42 and 0.46 for the Tinetti and MMSE, correspondingly, without the pre-shunt scores.

The weights of the predictors in the LASSO model were examined for their contributions in the prediction. The features selected in predicting Tinetti primarily involved the ventricle and sulci, such as the right parietal and frontal sulci and bilateral inferior LV (Fig. 4c). Noticeably, the enlargement of bilateral inferior LV was a strong predictor of low Tinetti score. The regions contributing to predicting MMSE showed a different pattern (Fig. 4f), which predominantly covered the cortical gyri and WM, such as the left angular gyrus, right cuneus, left fornix/stria terminalis, and left anterior deep WM. In addition, the regions that played roles in the prognostic test had overlap with those in the diagnostic test (Fig. 3), e.g., the inferior LV, fornix/stria terminalis, and posterior deep WM, although different patients were involved in the two tasks.

## Discussion

This study investigated the role of systematic volumetric analysis for the diagnosis of INPH shunt candidates as assessed by their response to CSF drainage and prediction of neurological outcomes after shunt placement. We achieved a high diagnostic accuracy of 0.94 in discriminating CSF drainage responders from non-responders in a cohort of probable INPH patients. The volumetric analysis also showed promising performance in predicting the gait and cognitive outcomes after shunt placement, with high correlations with the ground truth (*r* = 0.80–0.93).

Compared to the existing volumetric studies in INPH, our automated segmentation pipeline parcellates the brain into 283 structures, and the fine elements can be grouped into different granularity levels according to their ontological relationship, tailored to different studies. Our results demonstrated that the diagnostic and prognostic accuracy increased as the granularity level increased, illustrating the importance of fine segmentation. Another unique advantage is that our atlas definition was not only limited to GM and WM but also the ventricle and sulcal regions, e.g., 12 ventricular ROIs and 18 sulcal ROIs (Supplementary Table S1). This can be particularly useful for INPH studies, as the pattern of CSF distribution is an important marker of INPH [16, 17]. Note that image acquisition protocols slightly differed among patients, but it was unlikely to affect the results, as the segmentation pipeline has shown to be robust against protocols and highly reproducible in our previous studies [24, 36].

The fine structural segmentation, combined with the RFE model, led to a high classification accuracy in estimating the response to CSF drainage. Volumetric analysis has been used in the diagnosis of INPH in a handful of studies. Miskin et al [13] segmented the brains into GM, WM, ventricle, and hippocampus and reported an accuracy of 94% in the classification of the INPH patients and AD patients from the ADNI database. Serulle et al [14] performed coarse tissue segmentation and multiparametric model analysis and showed an overall accuracy of 96.3% accuracy in differentiating INPH from AD and healthy controls. Yamada et al [7] used ventricle and subarachnoid space volumes to predict CSF drainage response and reported an AUC of 0.768 in a group of suspected INPH patients. Here, we designed a relatively challenging task with a cohort of INPH-like patients with enlarged ventricles, elevated Evan’s index, and clinical manifestation of gait and cognitive declines. The diagnostic accuracy in discriminating CSF drainage responders reached an AUC of 0.94, suggesting that the volumetric markers can be potentially used instead of the invasive CSF drainage test for clinical decisions if validated further in other cohorts. Note that we performed a binary classification, but not prediction of the neurological scores as the patient responses at 2 h after CSF drainage were rather heterogeneous in terms of the improvement. Compared to the non-responders to CSF drainage, the responders manifested larger volumes of inferior LV, hippocampus, and several cortical gyri in fronto-orbital lobes, but smaller posterior/parietal WM. The selective expansion of inferior LV and relatively well-preserved hippocampus and cortical thickness were consistent with existing studies of INPH in comparison with other neurological diseases [15, 37, 38]. Here, the control group shared clinical and radiological similarities with the responders, and therefore, the selected anatomical features may be more specific to INPH pathology.

The use of brain MRI in predicting neurological outcome in post-surgical INPH was largely limited to qualitative or semi-quantitative assessments, such as the DESH scale [10, 19, 21]. For example, Shinoda et al showed that the DESH score was correlated with the changes in the modified Rankin scale (a common outcome measure for stroke [39]) with *r* = - 0.79 [19]; Virhammar reported that the callosal angle was significantly smaller in patients who had favorite responses to shunt surgery than that in non-responders (59° versus 68°) [20] and that the odds ratio for the callosal angle, DESH, and temporal horn appeared to be significant for shunt outcome [40]; Ahmed et al showed that the neurological outcome after shunting did not differ for patients with or without the presence of DESH feature in their pre-shunting MRI [41]. We showed that whole-brain volumetric analysis, combined with the LASSO regression model, could be used to predict the gait and cognitive performance. The relatively high prediction accuracy (*r* = 0.80 and 0.88) supports the feasibility of using pre-surgical MRI to estimate patient outcomes. The features associated with the prediction showed distinctive patterns for Tinetti and MMSE. While the ventricle and sulci played essential roles in predicting Tinetti, the cortical gyri and WM regions were selected for MMSE prediction, indicating the gait and cognitive outcomes may be associated with different brain regions. This finding agreed with previous findings that CSF-based anatomical features were more effective in predicting gait outcomes than predicting cognitive outcomes [10, 19, 21].

There are several limitations in the current study. Due to the limited patient number in cohort 2, we could not use the volumetric analysis at the finest segmentation as the number of volumetric features far exceeded the sample size. A larger cohort would allow a more flexible analysis and higher prediction accuracy. Also, the current study only retrospectively included patients from a single site with a homogenized imaging protocol. Future prospective multi-center studies are needed to test the generalizability and accuracy of the proposed method. Moreover, the current study only concerned brain volumes, and morphological features, such as the callosal convexity and DESH, were not assessed or compared. Future work that incorporates the volumetric and shape information into the feature analysis may further improve the prediction accuracy. Also, a combination of the morphological analysis with advanced imaging techniques, such as phase-contrast-based CSF flow imaging [9], diffusion MRI, and MRI spectroscopy [42], may further improve the diagnostic and prognostic values of brain MRI in the clinical management of INPH. On the other hand, the easy accessibility of 3D T1-weighted MRI enhanced the clinical translatability of the proposed approach.

In summary, we demonstrated that systematic volumetric analysis with fine segmentation could reliably differentiate responders to CSF drainage test and predict the neurological outcomes after shunt placement in INPH patients. The high-performance diagnostic tool with cloud-based multi-granularity segmentation could be readily integrated into clinical routine.

## Supplementary information

ESM 1 (DOCX 23 kb)

## Abbreviations

AD: Alzheimer’s disease
CSF: Cerebrospinal fluid
INPH: Idiopathic normal pressure hydrocephalus
LASSO: Least absolute shrinkage and selection operator
LV: Lateral ventricle
MMSE: Mini-Mental State Examination
RFE: Recursive feature elimination
ROC: Receiver operator curve
SVM: Support vector machine
TUG: Timed up and go
