# Supplementary material for: Systematic volumetric analysis predicts response to CSF drainage and outcome to shunt surgery in idiopathic normal pressure hydrocephalus
Source: Eur Radiol. 2021 Jan 3;31(7):4972–80. doi: 10.1007/s00330-020-07531-z (PMC8213563; doi:10.1007/s00330-020-07531-z)
Supplement: Supplementary file 1 — (DOCX 23 kb) [file 330_2020_7531_MOESM1_ESM.docx]

**Supplementary Materials**

**Table S1**: 283 regions of interest (ROIs) defined in the atlas at the finest segmentation, including 92 GM ROIs, 114 WM ROIs, 12 ventricular ROIs, 18 sulci ROIs, 34 brainstem and cerebellar ROIs, and 13 junk labels as place holders.

| **Gray matter (L/R)** | **White matter (L/R)** | **Ventricles** |
| --- | --- | --- |
| Superior frontal gyrus | Anterior corona radiata | Anterior lateral ventricle (L) |
| superior frontal gyrus/ prefrontal cortex | Superior corona radiata | Anterior lateral ventricle (R) |
| superior frontal gyrus/ pole | Posterior corona radiata | Body of the lateral ventricle (L) |
| Middle frontal gyrus | Genu of corpus callosum | Body of the lateral ventricle (R) |
| Middle frontal gyrus (dorsolateral prefrontal cortex) | Body of corpus callosum | Atrium of the lateral ventricle (L) |
| Inferior frontal gyrus/pars opercularis | Splenium of corpus callosum | Atrium of the lateral ventricle (R) |
| Inferior frontal gyrus/pars orbitalis | Lateral part of the periventricular white matter | Occipital lateral ventricle (L) |
| Inferior frontal gyrus/ pars triangularis | Anterior limb of internal capsule | Occipital lateral ventricle (R) |
| Lateral fronto-orbital gyrus | Posterior limb of internal capsule | Inferior lateral ventricle (L) |
| Middle fronto-orbital gyrus | Retrolenticular part of internal capsule | Inferior lateral ventricle (R) |
| Gyrus rectus | External capsule | III_ventricle |
| Postcentral gyrus | Cingulum (cingulate gyrus part) | IV_ventricle |
| Precentral gyrus | Cingulum (hippocampal part) |  |
| Superior parietal lobule | Fornix/stria terminalis | **Sulci (L/R)** |
| Supramarginal Gyrus | Fornix | Central sulcus |
| Angular gyrus | Fimbria | Sylvian fissure and anterior insular sulcus |
| Precuneus | Inferior fronto-occipital fasciculus | Sylvian fissure and posterior insular sulcus |
| Superior temporal gyrus | Posterior thalamic radiation | Extension of Sylvian fissure into supramarginal gyrus |
| Superior temporal gyrus/ pole | Sagittal stratum | Sulci of the frontal lobe |
| Middle temporal gyrus | Superior fronto-occipital fascicul | Sulci of the parietal lobe |
| Middle temporal gyrus_pole | Superior longitudinal fasciculus | Sulci of the cingulate gyrus |
| Inferior temporal gyrus | Anterior Commissure | Sulci of the occipital lobe |
| Parahippocampal gyrus | sWM of the superior frontal gyrus | Sulci of the temporal lobe |
| Entorhinal area | sWM of the superior frontal gyrus/ prefrontal cortex |  |
| Fusiform gyrus | sWM of the superior frontal gyrus/ pole | **Brainstem and cerebellum** |
| Superior occipital gyrus | sWM of the middle frontal gyrus | Midbrain |
| Middle occipital gyrus | sWM of the middle frontal gyrus/ dorsolateral prefrontal cortex | Pons |
| Inferior occipital gyrus | sWM of the inferior frontal gyrus/pars opecularis | Medulla |
| Cuneus | sWM of the inferior frontal gyrus/pars orbitalis | Medial lemniscus |
| Lingual gyrus | sWM of the inferior frontal gyrus/pars triangularis | Mammillary body |
| Rostral_Anterior cingulate cortex | sWM of the lateral fronto-orbital gyrus | Cerebral peduncle |
| Subcallosal_Anterior cingulate cortex | sWM of the middle fronto-orbital gyrus | Inferior cerebellar peduncle/pons |
| Subgenual anterior cingulate cortex | sWM of the gyrus rectus | Corticospinal tract |
| Dorsal Anterior cingulate cortex | sWM of the postcentral gyrus | Superior cerebellar peduncle |
| Posterior cingulate cortex | sWM of the precentral gyrus | Middle cerebellar peduncle |
| Insula | sWM of the superior parietal lobule | Pontine crossing tract |
| Amygdala | sWM of the supramarginal gyrus | Inferior cerebellar peduncle |
| Hippocampus | Angular white matter | Claustrum (left) |
| Caudate nucleus | sWM of the precuneus | Cerebellum gray matter |
| Putamen | sWM of the superior temporal gyrus | Cerebellum white matter |
| Globus Pallidus | sWM of the superior temporal gyrus/ pole | Middle cerebellar peduncle in the cerebellum |
| Thalamus | sWM of the middle temporal gyrus | Inferior cerebellar peduncle in the cerebellum |
| Hypothalamus | sWM of the middle temporal gyrus/ pole |  |
| Nucleus accumbens | sWM of the inferior temporal gyrus |  |
| Red nucleus | sWM of the fusiform gyrus |  |
| Substantia nigra | sWM of the superior occipital gyrus |  |
|  | sWM of the middle occiptial gyrus |  |
|  | sWM of the inferior occipital gyrus |  |
|  | sWM of the cuneus |  |
|  | sWM of the lingual gyrus |  |
|  | sWM of the rostral anterior cingulate cortex |  |
|  | sWM of the subcallosal anterior cingulate cortex |  |
|  | sWM of the subgenual anterior cingulate cortex |  |
|  | sWM of the dorsal anterior cingulate cortex |  |
|  | sWM of the postrior cingulate cortex |  |
|  | Anterior peri-ventricular WM |  |
|  | Posterior peri-ventricular WM |  |

Abbreviations: subcortical white matter –sWM.

**Table S2**: Brain structures that contributed to the classification of CSF-drainage responders and non-responders, along with their weights in the REF model. 5, 16, and 79 ROIs were selected at levels 3, 4, and 5, respectively. At level 5, only the top 20 ROIs with the highest absolute weights were listed.

| Level 3 | Weight | Level 4 | Weight | Level 5 | Weight |
| --- | --- | --- | --- | --- | --- |
| LV (L) | 0.221 | Inferior LV (R) | 0.183 | Inferior frontal WM /pars triangularis (R) | 0.133 |
| Medulla (R) | 0.192 | Fornix/stria terminalis (R) | 0.161 | Inferior frontal gyrus /pars triangularis (R) | 0.118 |
| 3^rd^ ventricle | 0.178 | Fornix/stria terminalis (L) | 0.114 | Superior frontal gyrus (R) | 0.115 |
| Basal ganglia (R) | 0.169 | Orbital gyrus (R) | 0.148 | Fornix/stria terminalis (L) | 0.097 |
| Occipital sulci (L) | -0.153 | Orbital gyrus (L) | 0.115 | Fornix/stria terminalis (R) | 0.095 |
|  |  | Superior frontal gyrus (R) | 0.128 | Inferior frontal WM /pars opercularis (L) | 0.088 |
|  |  | Inferior frontal gyrus (R) | 0.122 | Superior frontal WM (R) | 0.087 |
|  |  | CGH_R | 0.115 | Inferior LV (R) | 0.087 |
|  |  | Hippocampus (R) | 0.112 | Inferior frontal gyrus /pars orbitalis (R) | 0.086 |
|  |  | Posterior deep WM (L) | -0.193 | Middle temporal gyrus (R) | 0.085 |
|  |  | Postcentral gyrus (R) | -0.161 | Middle fronto-orbital WM (right) | 0.082 |
|  |  | Supramarginal gyrus (R) | -0.134 | Postcentral gyrus (R) | -0.124 |
|  |  | Superior parietal gyrus (L) | -0.120 | Superior parietal gyrus (L) | -0.115 |
|  |  | Parietal WM (R) | -0.127 | Inferior frontal gyrus /pars opercularis (R) | -0.101 |
|  |  | Parietal WM (L) | -0.118 | Posterior corona radiata (L) | -0.093 |
|  |  |  |  | Supramarginal WM (R) | -0.093 |
|  |  |  |  | Pontine crossing tract (L) | -0.083 |
|  |  |  |  | Precentral WM (L) | -0.082 |
|  |  |  |  | Cuneus WM of (L) | -0.081 |
|  |  |  |  | Supramarginal gyrus (R) | -0.081 |
